# A standardized framework to evaluate the quality of studies using TDABC in healthcare: the TDABC in Healthcare Consortium Consensus Statement

**DOI:** 10.1186/s12913-020-05869-0

**Published:** 2020-12-01

**Authors:** Ana Paula Beck da Silva Etges, Carisi Anne Polanczyk, Richard D. Urman

**Affiliations:** 1 National Institute of Science and Technology for Health Technology Assessment (IATS)- CNPq/Brazil (project: 465518/2014-1), Ramiro Barcelos 2350, Porto Alegre, Brazil; 2 School of Technology, Pontifícia Universidade Católica do Rio Grande do Sul, Porto Alegre, Brazil; 3 Programa de Pós-graduação em Epidemiologia da Escola de Medicina da Universidade Federal do Rio Grande do Sul, Porto Alegre, RS Brazil; 4 http://www.tdabcconsortium.com/; 5 Hospital Moinhos de Vento, Porto Alegre, Brazil; 6 Department of Anesthesiology, Perioperative and Pain Medicine, Brigham and Women’s Hospital/Harvard Medical School, Boston, MA USA; 7 Center for Perioperative Research, Brigham and Women’s Hospital, Boston, MA USA

**Keywords:** Time-driven activity based costing, Value-based healthcare, TDABC, VBHC, Microcosting, Healthcare costs

## Abstract

**Background:**

This Consensus Statement introduces a standardized framework, in a checklist format, to support future development and reporting of TDABC studies in healthcare, and to encourage their reproducibility. Additionally, it establishes the first formal networking of TDABC researchers through the creation of the TDABC in Healthcare Consortium.

**Methods:**

A consensus group of researchers reviewed the most relevant TDABC studies available in Medline and Scopus databases to identify the initial elements of the checklist. Using a Focus Group process, each element received a recommendation regarding where in the scientific article section it should be placed and whether the element was required or suggested. A questionnaire was circulated with expert researchers in the field to provide additional recommendations regarding the content of the checklist and the strength of recommendation for each included element.

**Results:**

The TDABC standardized framework includes 32 elements, provides recommendations where in the scientific article to include each element, and comments on the strength of each recommendation. All 32 elements were validated, with 21 elements classified as mandatory and 11 as suggested but not mandatory.

**Conclusions:**

This is the first standardized framework to support the development and reporting of TDABC research in healthcare and to stablish a community of experts in TDABC methodology. We expect that it can contribute to scale strategies that would result in cost-savings outcomes and in value-oriented strategies that can be adopted in healthcare systems and institutions.

**Supplementary Information:**

The online version contains supplementary material available at 10.1186/s12913-020-05869-0.

## Background

The use of Time-Driven Activity-Based Costing (TDABC) methodology to evaluate costs in healthcare has expanded in the last 5 years, with studies encompassing different healthcare fields such as surgery and perioperative care [1, 2], cancer [3], transplants [4, 5], outpatient care [6], psychiatry [7], and hospital management [8] as reported in recent systematic reviews [9, 10]. The increasing number of TDABC studies suggests that this is an essential method for supporting value-based healthcare management programs [11–13]. In fact, in 2020 the Society for Perioperative Assessment and Quality Improvement (SPAQI) outlined eight principles of TDABC and argued that the aforementioned method provides cost transparency to demonstrate value in perioperative care [14]. Even though the use of TDABC has advanced, its application in microcosting studies in healthcare presents methodological heterogeneity and demonstrates differences in how researchers design studies and report their methods and results [9, 15].

The International Society for Pharmacoeconomic and Outcomes Research (ISPOR) recently revised the Consolidated Health Economic Evaluation Reporting Standards (CHEERS) statement, which provides recommendations to optimize the reporting of health economic evaluations [16]. The CHEERS statement provides a list of 24 items that a scientific article on economic evaluation should contain. However, specific items in the list that guide cost reporting, i.e. “estimating resources and costs (13a and 13b)” and “Incremental costs and outcomes (19)” lack detailed methods to achieve high quality and real individual cost information. The use of bottom-up microcosting techniques are described in the literature as the “gold-standard” recommendation to evaluate the cost information in economic analyses [17, 18], and the TDABC methodology can be used to better perform the microcosting studies [9, 15].

This Consensus Statement aims to provide a standardized framework, in a checklist format, to support future development and reporting of TDABC studies in healthcare. This checklist is not intended to be a rigid guideline, but rather as a tool to assist researchers when designing and reporting TDABC studies. It should prompt reviewers and readers to question the lack or appropriateness of certain elements when developing a healthcare-related TDABC project.

Our overall goal is to encourage reproducibility in TDABC studies, while taking into account the different variables that may influence cost analysis and the different ways in which TDABC has been implemented in microcosting studies around the world. In addition, this Consensus Statement aims to establish a global community of TDABC researchers through the creation of the TDABC for Healthare Consortium (www.tdabcconsortium.com).

## Methods

**Fig. 1:**
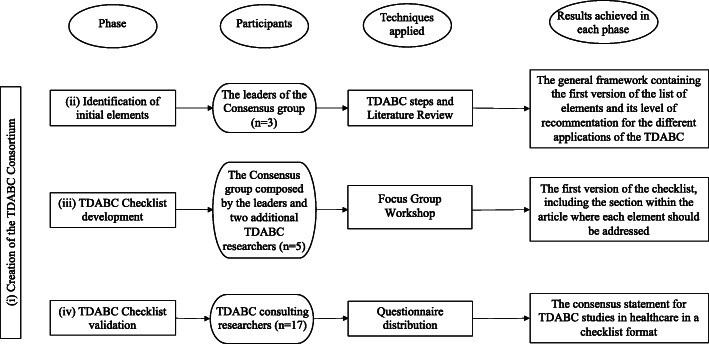
Sequence of methodological steps followed to develop a standardized framework consensus to evaluate the quality of studies using TDABC in healthcare

The overall methodology was based, at least in part, on other recent checklists used for reporting study results [19]. Our approach consisted of four phases: (i) Selection of TDABC expert researchers and identification of initial elements of the checklist; (ii) Application of a Focus Group process to build the checklist [15]; (iii) Selection of external opinions to validate the checklist; (iv) Creation of the checklist. Each phase involved different researchers who were categorized into the following groups: leaders of the consensus group (3 researchers: A.P.E., C.A.P., and R.D.U.); the consensus group (3 leaders and two additional researchers); and the TDABC consulting researchers (17 researchers invited to answer the questionnaire). Therefore, during the checklist development process, a total of 22 experts including researchers, clinicians and healthcare managers were involved. Figure 1 is a flowchart which details the sequence of consensus phases, the types of participants who were involved in each phase, techniques applied, and the results following each phase. Each of the four phases is described in more detail below.

### Selection of TDABC expert researchers and identification of initial elements of the checklist

The authors of this article represent the leaders of the consensus group, responsible for the design and management of the checklist. The TDABC methodology [19] and the 8-step framework applied in TDABC microcosting studies [4] were used as guides to create the checklist element sequence. The checklist element sequence was created by posing a question to the participant which required a binary answer (“yes” or “no”). This approach was used to help future checklist users to assess each element when reviewing a TDABC in healthcare study.

Next, the 15 most cited cost analyses or management studies that detailed opportunities in applying TDABC in healthcare, available in Scopus database, were reviewed to identify the presence of each element in the studies. The keywords “Time-Driven Activity-Based Costing” OR “TDABC” and “Health” were used to search for the articles. Studies with a high citation but that only reported the TDABC as a cost analysis strategy used while not detailing its application, were not considered (10 articles – see Additional file 1). Those articles commented on the method theoretically but did not apply it in a real-world setting. The articles used in this Consensus statement contained at least 21 citations. Each element in the articles was categorized, where “Required” was attributed to the elements encountered in all studies, and “suggested” attributed for elements encountered in only a few studies.

Since a few studies aimed to assess costs or compare the TDABC with traditional cost methods (Type I) [20–22] and others aimed to apply TDABC to support healthcare service management (Type II) [23–25], the elements were further classified as follows: (#) for elements suggested by all studies; (*) for cost assessment (Type I); and (#*) for management (Type II).

### Application focus group process to build the checklist

The consensus group, formed by the project leaders and two additional researchers with significant experience in TDABC, evaluated the framework in a Focus Group section. The workshop was organized to develop and clarify the elements in the checklist and to define the best section in a research study to place each element. The group followed the sequence of reiterative steps suggested by existing Focus Group methodology for health and medical research [26]. The moderator (A.P.E.) used the researchers’ current experiences from different projects that are using TDABC to evaluate hospital costs of the bone marrow transplant treatment [4] and a telemedicine service [27] in Brazil, to facilitate the review and discussion of each element.

For the Focus Group workshop, first the moderator explained the goals of the checklist and presented the proposed elements. Next, each researcher was invited to: classify the level of importance of each element using a 5-point scale for rating [28] (1- it is not important and 5 – it is extremely important); identify if the element was important for all studies, or specific to Type I (studies that applied the TDABC only to assess costs) or Type II (studies that applied the TDABC to support healthcare service management); and identify the most appropriate section in the publication to place each element. Researchers received 1 h to individually complete the first stage. Subsequently, a discussion section using the experiences from the bone marrow transplant [4] and telemedicine [27] projects was carried out by a moderator who encouraged the interaction without creating bias [29]. For any disagreement among members, the researchers discussed their areas of disagreement and reached a consensus based on the examples that were encountered during the research group members’ recent experiences with TDABC studies [4, 5, 27] and the results encountered in the recent systematic review developed by the leaders of the consensus group [15].

With the results generated in the workshop section, the consensus group then discussed the framework to incorporate additional best practice elements.

### Selection of external opinions to validate the checklist

The TDABC study checklist was distributed to authors of prior key TDABC studies, which were identified in our most recent systematic review [15] exploring TDABC in healthcare as well as to authors who frequently published studies using this methodology. A total of 17 published authors included researchers with Master’s and Doctoral degrees from North and South America, Europe, Oceania and Africa who collaborated by answering a Google Forms questionnaire (Appendix). The questionnaire contained five possible responses to define whether an element fell into one of the following categories: (i) Mandatory; (ii) Strongly suggested, but not mandatory; (iii) Suggested; (iv) Optional; (5) Do not include. The interviewees were invited to answer the questionnaire and consented to participate by having their opinions provided in the questionnaire to be included in the research analysis.

### Creation of the checklist

The answers were combined into a spreadsheet by the consensus group for data analysis. The frequency for each selected answer was used as the criterion to define the strength of recommendation for each element, so that the strength of recommendation suggested by the majority of those interviewed regarding each element was used to define the final strength of recommendation. A final framework including 32 elements, the strength of each recommendation, and the location within the article where each element should be addressed, was used to generate the final framework version to evaluate the quality of studies using TDABC.

The researchers involved in all phases of this project represent the initial members of the newly-formed TDABC in Healthcare Consortium (www.tdabcconsotium.com). The Consortium is composed of a collaborative group of researchers and managers who are working to improve the quality of projects that apply TDABC in healthcare and disseminate methodological advances for TDABC to promote value-based healthcare (VBHC).

## Results

### Selection of TDABC expert researchers and identification of initial elements of the checklist

The general framework containing elements to guide the reporting of TDABC in healthcare studies includes the revised literature results used to classify the elements (Table 1). The first column depicts the TDABC steps, the second contains the elements, and the third indicates if the element is classified as “required” or “suggested”. The symbol (eg. #, *, #*) coding indicates if the element is recommended for all studies (#), only for Type I (*) or only Type II (#*).

**Table 1:** List of elements for reporting TDABC studies as identified by the consensus group

TDABC Steps	Essential elements	Classification	Demeere et al. (2009) [30]	Laviana et al. (2016) [31]	Akhavan et al. (2016) [32]	Mc Laughlin et al. (2014) [33]	Kaplan (2014) [34]	Donovan et al. (2014) [35]]	French et al. (2013) [39]	Kaplan AL et al. (2014) [40]	Kaplan (2014) [24]	Tseng at al. (2018) [41]	Bauer-Nilsen (2017) [42]	Yu et al. (2016) [43]	Chen et al. (2015) [44]	Thaker et al. (2016) [45]	Erhun et al. (2017) [46]
Citation (Scopus)			89	75	67	43	92	32	32	34	35	27	25	21	24	21	24
1 -Identify the study question or technologies to be assessed	(#) Are the results are being explored for general health service management or redesign and value or only to assess costs?	Required	General health service management or redesign	General health service management or redesign	Assessing costs and compare with traditional method	General health service management or redesign	General health service management or redesign	Assess costs and compare with traditional method	General health service management or redesign	General health service management or redesign	General health service management or redesign	General health service management or redesign	Assessing costs and compare with traditional method	Assessing costs and compare with traditional method	Assessing costs and compare with traditional method	Building a value framework	General health service management or redesign
	(#) Is the clinical pathway, technology or procedure studied justified because of an interest from government, hospital, society or a Health Technology Assessment analysis?	Required	Yes - hospital/clinic	Yes, hospital	Yes, hospital	Yes, hospital	Yes, hospital	Yes, hospital	Yes, hospital	Yes, hospital	Yes, hospital	Yes, hospital	Yes, hospital	Yes, hospital	Yes, hospital	Yes, society	Yes, hospital
	(#) Is the TDABC method selection being justified?	Required	Yes	Yes	Yes	Yes	Yes	Yes	Yes	Yes	Yes	Yes	Yes	Yes	Yes	Yes	Yes
2 - Map the process: the care-delivery value chain	(#) Are authors using specific methodologies to design the care pathway?	Suggested	No	No	No	Yes, BPMN, Delphi	Yes, Industrial engineering expertise	No	No	Yes, Industrial engineering expertise	No	Yes, Industrial engineering expertise	No	Yes, Lean and Six Sigma	No	No	No
	(#) Are authors exploring a multidisciplinary team to apply the TDABC? (Design the process, correctly consider clinical characteristics, correctly evaluate costs)	Required	Yes	Yes	Yes	Yes	Yes	Yes	Yes	Yes	Yes	Yes	Yes	Yes	Yes	Yes	yes
	(#) Are authors reporting activities in the process map on a macro level?	Required	Yes	Yes	Yes	Yes	Yes	Yes	Yes	Yes	Yes	Yes	Yes	Yes	Yes	Yes	Yes
	(#) Are authors reporting activities in the process map on a micro level?	Suggested	No	Yes	Yes	No	Yes	No	No	No	Yes	Yes	No	Yes	No	Yes	No
	(#) Is the full process map (or a part of) being presented in a picture or graphic display?	Suggested	Yes	Yes	Yes	Yes	Yes	No	Yes	Yes	Yes	Yes	Yes	Yes	Yes	Yes	Yes
3 - Identify the main resources used in each activity and department	(#) Is a table or a map being presented to illustrate the association between activities and resources?	Suggested	Yes	No	No	Yes	No	No	No	Yes	Yes	Yes	No	Yes	No	No	Yes
	(#)Are authors reporting an observation in-situ approach to better identify resources used in each activity?	Required	Yes	Yes	Yes	Yes	Yes	Yes	Yes	Yes	Yes	Yes	Yes	Yes	Yes	Yes	Yes
	(#) Are authors interviewing the professionals to better identify resources used in each activity?	Required	Yes	Yes	Yes	Yes	Yes	Yes	Yes	Yes	Yes	Yes	Yes	Yes	Yes	Yes	Yes
4 - Estimate the total cost of each resource group and department	(#) When using hospital financial database, it is being explained how the finance department was involved to access data?	Suggested	No	No	No	No	Yes	Yes	Yes	Yes	Yes	Yes	No	Yes	No	No	Yes
	(#) When using external financial databases, is it being defined which one and how data were accessed?	Required when authors are using national financial databases	NA	NA	NA	NA	NA	Medicare	NA	NA	NA	NA	Medicare	Combined salary references from different Medical Associations	NHS	NA	NA
	(#) When mixed financial database are being used (for example, salaries from external reference and structural costs from the hospital) is the source of each data variable being stated?	Suggested	NA	NA	NA	NA	NA	Yes	NA	NA	NA	NA	Yes	Yes	Yes	NA	NA
	(#) Did the authors explain how the overhead costs are being considered?	Suggested	No	No	Yes	Yes	Yes	Yes	No	No	Yes	Yes	No	No	No, it is being assessed only direct costs	No	No
5 - Estimate the capacity of each resource and calculate capacity cost rate (CCR-$/h)	(#) Are authors defining if the capacity data used represents the total capacity per resource or it is being considered an expected idleness?	Suggested	Yes, an expected idleness was considered	Yes, an expected idleness was considered	Yes, an expected idleness was considered	Yes, an expected idleness was considered	Yes, an expected idleness was considered	Yes, an expected idleness was considered	Yes, an expected idleness was considered	Yes, an expected idleness was considered	Yes, an expected idleness was considered	no	No	No	No	No	Yes, an expected idleness was considered
	(#) When authors are considering an expected idleness, it is being explained how real performance data were collected and analyzed?	Suggested	No	No	No	No	Yes	No	No	No	No	NA	NA	NA	NA	NA	Yes
6 - Analyze the time estimates for each resource used in an activity	(#) Are authors explaining how time data was collected?	Suggested	Yes	Yes	Yes	Yes	No	Yes	Yes	Yes	Yes	yes	Yes	Yes	No	Yes	Yes
	(#) Are authors using interviews with professionals in addition to medical record review to estimate time data?	Suggested	No	Yes	Yes	Yes	No	Yes	No	Yes	Yes	yes	Yes	Yes	No	Yes	Yes
	(#) When using chronanalysis, is it being explained how the sample of data was defined?	Required	Yes - defined a sample	Yes - defined a sample	NA	NA	NA	NA	NA	NA	NA	NA	NA	NA	No	Yes, defined sample	No
	(#) Is digital technology being used to collect real time data, such as mobile app, wearable, drone, etc.?	Suggested	No	No	No	No	No	No	No	No	No	No	No	No	No	No	No
7 - Calculate the total cost of patient care and cost-data analytics	(#) Is the median or average cost per patient (or per technology) being calculated?	Required	Yes	Yes	Yes	No	It was not presented cost results; the paper is focused on express the cost-saving opportunities identified	No, the article focuses the discussion in compare TDABC with RVU cost methods.	Yes	Yes	No, the paper explores how TDABC can be used to increase value, orient redesign actions and decrease costs without impact outcomes.	Yes	Yes	Yes	Yes	Yes	The paper is a methodological paper to orient process as costs comparisons between centers, but do not details cost results.
	(*) Are authors presenting the cost per each patient included in the sample? (Chart bar, table, etc.)?	Suggested	No	No	No	no			No	No		No	No	No	No	No	
	(*) Is the median or average cost per activity on a macro level being presented?	Required	Yes	Yes	Yes	Yes			Yes	Yes		Yes	Yes	Yes	Yes	Yes	
	(*) Is the median or average cost per activity on a micro level being presented?	Suggested	No	No	No	No			No	No		No	No	No	No	No	
	(#*) Is the median or average cost per resource being presented?	Required	No	No	Yes	No			No	Yes		No	No	No	Yes	No	
	(*) Are authors exploring statistical analyses to better understand costs along the process of care?	Suggested	No	No	No	No			Yes	No		No	No	No	No	Yes	
	(#*) If the objective was to use the study to support management and value decisions, are authors reporting how value increasing was achieved or if they are planning to achieve it?	Required	Yes	Yes	NA	Yes	Yes	Yes	Yes	NA	Yes	Yes	NA	NA	NA	Yes	Yes

The results shown in Table 1 were the elements identified in all reviewed studies such as the justification for the use of TDABC, the involvement of a multidisciplinary team, the use of formal interviews and observations in situ, the representation of the patient flow at a macro level in a process map, and the connection with value in healthcare decisions. On the other hand, we identified significant heterogeneity in how the TDABC methodological steps were being followed in the studies, such as the patient flow map design, the financial data used (hospital, or external databases) and its level of detail, the inclusion of overhead costs, ways to estimate capacities, time data collection, and approaches used to report the results.

### Application of focus group process to build the checklist

By applying the Focus Group process in the first round, we identified two different answers at a level of 2 point-difference and eight different answers at a level of 1 point-difference to confirm the level of importance of each element. We also determined the section within the published study where each element should be placed and if the placement was found in “All studies” (#), only Type I (*), or only Type II (#*). We reached the consensus for both of these questions after the first round. The divergencies of opinion observed during the first round regarding the level of importance of each item were discussed and, in the second round, a consensus was reached for all of them. Finally, suggestions of additional elements that should be included were discussed. The ones agreed upon by the consensus group were included in the checklist (bold letters in Tables 2 and 3). The TDABC elements, differences in opinion, the final consensus reached about level of importance, Type category and suggested paper section are shown in Table 2.

**Table 2:** The checklist with TDABC elements after the Delphi workshop

TDABC elements	Paper section	Researcher A	Researcher B	Consensus
(#) 1.1 It is defined if the results are being explored for general health service management or redesign and value or only to assess costs?	Introduction	5	5	5
(#)1.2 Is the clinical pathway, technology or procedure studied justified because of an interest from government, hospital, society or a Health Technology Assessment analysis?	Introduction	5	5	5
**(#)1.3 Are study limitations being presented?**	Discussion	Element suggested by the participants of the Delphi workshop		5
(#)1.4 Is the TDABC method selection being justified?	Introduction	5	3	3
(#) 2.1 Are authors using specific methodologies to design the care pathway?	Methods	3	3	3
(#) 2.2 Are authors using a multidisciplinary team to apply the TDABC? (Design the process, correctly consider clinical characteristics, correctly evaluate costs.)	Methods	5	5	5
(#) 2.3 Are authors reporting activities in the process map on a macro level?	Methods	5	5	5
(#) 2.4 Are authors reporting activities in the process map on a micro level?	Methods	2	2	2
(#) 2.5 Is the full process map (or a part of) being presented in a picture or graphic display?	Results	3	4	4
(#) 3.1 Is a table or a map being presented to illustrate the association between activities and resources?	Results	3	4	3
**(#) 3.2 Are resources that are included in the analysis being defined and justified?**	Methods	Element suggested by the participants of the Delphi workshop		3
(#) 3.3 Are authors reporting observation in-situ approach to better identify resources used in each activity?	Results	5	3	4
(#) 3.4 Are authors interviewing the professionals to better identify resources used in each activity?	Results	5	4	4
(#) 4.1 When using hospital financial database, it is being stated how those data were collected and analyzed?	Methods	2	2	2
**(#) 4.2 Are authors defining the currency and applying discount taxes when it is necessary?**	Methods	Element suggested by the participants of the Delphi workshop		4
(#) 4.3 When using external financial databases, is there a description of the database and how those data were accessed?	Methods	5	5	5
(#) 4.4 When mixed financial database data are being used (for example, salaries from external reference and structural costs from the hospital) is the origin of each data variable being stated?	Methods	5	4	4
(#) 4.5 Did the authors explain how the overhead costs are being considered?	Methods	3	3	3
(#) 5.1 Are authors defining if the capacity data used represents the total capacity per resource or it is being considered an expected idleness?	Methods	5	5	5
(#) 5.2 When authors are considering an expected idleness, it is explained how actual performance data were collected and analyzed?	Methods	3	4	3
(#) 6.1 Do authors explain how time data were collected?	Methods	5	5	5
(#) 6.2 Are authors using interviews with professionals crossed with medical record review to estimate time data?	Methods	5	4	4
**(#) 6.3 When using chronanalysis, it is being explained how the sample of data was defined?**	Methods	Element suggested by the participants of the Delphi workshop		5
**(#) 6.4 Is it being explained if the chronanalysis used digital technology to collect real time data, such as a mobile app, wearable, drone, etc.?**	Methods	Element suggested by the participants of the Delphi workshop		4
(#) 7.1 Is the median or average cost per patient (or per technology) being calculated?	Result and Discussion	5	5	5
(*) 7.2 Are authors presenting the cost per each patient that is included in the sample? (Chart bar, table, etc.)?	Result and Discussion	3	3	3
(*) 7.3 Is the median or average cost per activity on a macro level being presented?	Result and Discussion	5	5	5
(*) 7.4 Is the median or average cost per activity on a micro level being presented?	Result and Discussion	3	3	3
(*) 7.5 Is the median or average cost per resource being presented?	Result and Discussion	3	4	3
(#*) 7.6 Are the authors performing capacity idleness analysis?	Result and Discussion	Element suggested by the participants of the Delphi workshop		2
(*) 7.7 Are the authors exploring statistical analyses to better understand costs along the process of care?	Result and Discussion	3	3	3
(#*) 7.8 If the objective was to use the study to support management and value decisions, are authors reporting how value increasing was achieved or if they are planning to achieve it?	Result and Discussion	5	5	5

The column ‘Researcher A’ contains the answers attributed by one of the additional researchers using the scale 1–5 (1- it is not important and 5 – it is extremely important). The column ‘Researcher B’ contains the answers attributed by the other additional researcher using the scale 1–5. The columns consensus contains the final level of importance accorded between the two researchers and moderator.

**Table 3:** The consensus statement for TDABC studies in healthcare in a checklist format

TDABC elements	Mandatory	Strongly suggested, but not mandatory	Suggested	Option	Do not include	Classification	Paper section
(#) 1.1 It is defined if the results are being explored for general health service management or redesign and value or only to assess costs?	41%	53%	0%	0%	0%	**Strongly Suggested, but not mandatory**	**Introduction**
(#) 1.2 Is the clinical pathway, technology or procedure studied justified because of an interest from government, hospital, society or a Health Technology Assessment Analysis?	29%	35%	29%	6%	0%	**Mandatory**	**Introduction**
**(#) 1.3 Are study limitations being presented?**	94%	0%	0%	6%	0%	**Mandatory**	**Discussion**
(#) 1.4 Is the TDABC method selection being justified?	59%	12%	24%	6%	0%	**Mandatory**	**Introduction**
(#) 2.1 Are authors using specific methodologies to design the care pathway?	47%	24%	24%	6%	0%	**Mandatory**	**Methods**
(#) 2.2 Are authors using a multidisciplinary team to apply the TDABC? (Design the process, correctly consider clinical characteristics, correctly evaluate costs)	47%	35%	12%	6%	0%	**Mandatory**	**Methods**
(#) 2.3 Are authors reporting activities in the process map on a macro level?	59%	24%	6%	12%	0%	**Mandatory**	**Methods**
(#) 2.4 Are authors reporting activities in the process map on a micro level?	59%	12%	6%	12%	0%	**Mandatory**	**Methods**
(#) 2.5 Is the full process map (or a part of) being presented in a picture or graphic display?	35%	29%	24%	12%	0%	**Mandatory**	**Results**
(#) 3.1 Is a table or a map being presented to illustrate the association between activities and resources?	24%	47%	18%	12%	0%	**Strongly Suggested, but not mandatory**	**Results**
**(#) 3.2 Are the resources that are included in the analysis being defined and justified?**	41%	41%	18%	0%	0%	**Mandatory**	**Methods**
(#) 3.3 Are authors reporting observation in-situ approach to better identify resources used in each activity?	29%	41%	18%	12%	0%	**Mandatory**	**Results**
(#) 3.4 Are the authors interviewing the professionals to better identify resources used in each activity?	47%	35%	6%	6%	6%	**Mandatory**	**Results**
(#) 4.1 When using hospital financial database, it is being stated how those data were collected and analyzed?	47%	29%	18%	6%	0%	**Mandatory**	**Methods**
**(#) 4.2 Are authors defining the currency and applying discount taxes when it is necessary?**	29%	35%	24%	12%	0%	**Strongly Suggested, but not mandatory**	**Methods**
(#) 4.3 When using external financial databases, is there a description of the database and how those data were accessed?	41%	53%	0%	6%	0%	**Mandatory**	**Methods**
(#) 4.4 When mixed financial databases are being used (for example, salaries from external reference and structural costs from the hospital) is the origin of each data variable being stated?	53%	35%	12%	0%	0%	**Mandatory**	**Methods**
(#) 4.5 Did the authors explaining how the overhead costs are being considered?	53%	29%	12%	0%	6%	**Mandatory**	**Methods**
(#) 5.1 Are authors defining if the capacity data used represents the total capacity per resource or it is being considered an expected idleness?	41%	24%	24%	0%	12%	**Mandatory**	**Methods**
(#) 5.2 When authors are considering an expected idleness, it is explained how actual performance data were collected and analyzed?	29%	41%	12%	12%	6%	**Mandatory**	**Methods**
(#) 6.1 Are authors explaining how time data were collected?	76%	18%	6%	6%	0%	**Mandatory**	**Methods**
(#) 6.2 Are authors using interviews with professionals crossed with medical record review to estimate time data?	29%	53%	18%	0%	0%	**Strongly Suggested, but not mandatory**	**Methods**
**(#) 6.3 When using chronanalysis, it is being explained how the sample of data was defined?**	35%	35%	12%	6%	6%	**Mandatory**	**Methods**
**(#) 6.4 Is it being explained if the chronanalysis used a digital technology to collect real time data, such as mobile app, wearable, drone, etc.?**	12%	47%	29%	12%	0%	**Strongly Suggested, but not mandatory**	**Methods**
(#) 7.1 Is the median or average cost per patient (or per technology) being calculated?	65%	29%	0%	6%	0%	**Mandatory**	**Result and Discussion**
(*) 7.2 Are authors presenting the cost per each patient included in the sample? (Chart bar, table, etc.)?	41%	29%	24%	6%	0%	**Mandatory**	**Result and Discussion**
(*) 7.3 Is the median or average cost per activity on a macro level being presented?	53%	18%	29%	0%	0%	**Mandatory**	**Result and Discussion**
(*) 7.4 Is the median or average cost per activity on a micro level being presented?	29%	24%	24%	24%	0%	**Mandatory**	**Result and Discussion**
(*) 7.5 Is the median or average cost per resource being presented?	41%	35%	18%	6%	0%	**Mandatory**	**Result and Discussion**
(#*) 7.6 Are authors performing capacity idleness analysis?	18%	53%	12%	12%	6%	**Strongly Suggested, but not mandatory**	**Result and Discussion**
(*) 7.7 Are authors exploring statistical analyses to better understand costs along the process of care?	29%	47%	18%	6%	0%	**Strongly Suggested, but not mandatory**	**Result and Discussion**
(#*) 7.8 If the objective was to use the study to support management and value decisions, are authors reporting how value increasing was achieved or if they are planning to achieve it?	29%	53%	6%	12%	0%	**Strongly Suggested, but not mandatory**	**Result and Discussion**

The columns ‘Mandatory, Strongly suggested, but not mandatory, Suggested, Option and Do not include’ present the relative frequency of answers observed for each recommendation level. The column ‘Classification’ contains the final level of recommendation attributed for each element, and the ‘Paper Section’ column indicates which article section each element should be posted by the authors

Three elements, which were reported differently by previous authors, were considered of lower level of importance (level 2) during our consensus process. In contrast, while studies reviewed on Table 1 identified elements as common, at the workshop they were considered important (level “5”) by consensus. Additionally, it is important to point out that this methods section concentrates on where the majority of elements should be addressed (16 of 32 elements).

### Addition of external opinions and creation of the checklist

Based on the data obtained from the questionnaire, we confirmed the strength of the recommendation for each element in the framework that may be used to guide future TDABC in healthcare studies. Twenty-one elements were judged to be “Mandatory” and 11 elements were “Strongly Suggested but not Mandatory”. A few elements received one or two “Do not include” recommendations. However, we did not exclude the elements because of the “majorities’ opinion” criteria, which was used to define the strength of recommendation for each element. Table 3 shows the standardized TDABC framework to support the development and reporting of TDABC studies in healthcare.

All the elements that should be placed in the methods section were confirmed as mandatory, as was previously suggested by the level of importance registered in the Focus Group workshop, with only one exception for the element of currency and discount taxes. For this specific element, once the framework is applied to support multiregional or multi-period cost analyses, it is important to use the most current inflation information available on the world data bank website (https://data.worldbank.org/) to achieve accurate cost information and to compare costs. The following elements were all classified as mandatory and suggested to be placed in the methods section: patient flow map design, financial data used (hospital, or external databases) and its level of detail, the inclusion of overhead costs, the method used to estimate capacities, and the time data collection.

## Discussion

The principles of Value-based Healthcare (VBHC) include strategies for alleviating the burden of high expenses in healthcare [13, 30, 36]. However, for successful implementation of VBHC interventions, it is important to achieve accuracy on cost information [31]. This Consensus Statement is the first to introduce a standardized framework, in a checklist format, to support the development and reporting of TDABC studies in healthcare.

The use of advanced costing methods in healthcare offers an opportunity for researchers to fully explore alternatives to improve allocation of public healthcare resources [10]. The group’s recent systematic review about TDABC in the VBHC context identified 17 studies that used the TDABC to increase value in healthcare by assessing cost-savings throughout the optimization of the episode of care-cycle [15]. Even though the TDABC was already defined as the “gold standard” to assess real costs in healthcare, the application of TDABC in scientific studies showed enormous variability on how the methodology has been employed [9, 15]. To solve this problem, we developed the framework that consolidated methodological guidance for the application of TDABC. By following the framework, it is expected that those who design the studies and interpret the results can achieve better accuracy on cost information, understand limitations of some of these studies, and identify opportunities to reduce waste along the episodes of care, all contributing to the expansion of VBHC programs that include more precise cost outcomes assessment.

Neglecting certain costs or adopting inaccurate methodologies for cost estimations can introduce bias in interpretation of the results in health economic studies [32]. As a result of our Focus Group process, it is important to note the significance attributed to the proposed elements described in the methods section. The elements suggested to be posted in the given research study method section were classified as “Mandatory” and only one as “Strongly suggested, but not mandatory”. The researchers involved in the workshop process discussed the importance of detailed reporting of the essential elements to make the TDABC results valuable and replicable for future economic analyses, with the understanding that valid value-based comparisons are not possible without consensus around how costs were being calculated [32]. The strength of the recommendations regarding the methods elements based on the questionnaire responses highlights the importance of detailing of each step followed when applying the TDABC methodology. This guarantees the rigor of the TDABC use to assess costs (Type I) or to support healthcare service management (Type II).

One important issue when applying advanced cost methods is to define the scope, objective and business context where the study is being applied [33]. If the study is specific to assessing costs, the requirements can be more focused on financial aspects; on the other hand, once the method is being used to support healthcare service management, the results and discussion may explore the cost-savings and efficiency increase opportunities [37]. The identification of elements that are recommended for all TDABC studies (Type I and Type II) and others specific for Type I or Type II studies is the approach that we suggested in the tool to guide future authors to better consolidate results from their TDABC projects, considering the management and cost assessment or only the cost assessment objective.

In all studies reviewed to introduce the consensus elements, multidisciplinary teams were used to implement the TDABC, which suggests that the individual economic and financial backgrounds vary among the team members. The establishment of transparent communication when dealing with several backgrounds is not an easy task; the terms and definitions usually vary among physicians, engineers, economists, and others. The existence of a framework in a checklist format contributes to clarifying the communication among all team members. Once we have a better comprehension about the sequence of steps that may be followed in a TDABC project, we believe that the methodological rigor for applying the TDABC can be easily evaluated, contributing to the homogeneity of the studies and consequently to the reproducibility of results. This Consensus statement coincided with the creation of a global network of TDABC researchers, the TDABC Consortium, which can further contribute to the global dissemination of the standards of TDABC research reporting, the research that is still mostly concentrated in the USA [9, 15].

TDABC is valuable because of its detailed reporting on the episode of care, as it identifies how patients consume resources along the process of care. Therefore, if well-constructed, this method can be used to optimize the care cycle [31], [41], resulting in cost-saving outcomes [22]. If authors apply their TDABC strategies with higher methodological rigor, the results from parallel studies can be compared and replicated, opening new avenues to redesign initiatives to optimize care cycles in different healthcare settings [22]. We believe that by the achieving homogeneity and accuracy in studies that applied TDABC in healthcare, the cost information can be more efficiently used for health economic analysis to guide decisions about issues such as the incorporation of health technologies, economic evaluation of healthcare cycle redesign, healthcare system management, reimbursement policies evaluation, and the identification of waste reduction opportunities in clinical pathways.

The use of TDABC to identify benchmarks of care pathways is a valuable application of the method in the context of VBHC [15]. However, once the method is being used to evaluate or compare costs and care pathways internationally, authors must take into consideration the study’s economic assessment perspective and each healthcare system’s context before accepting conclusions about costs and efficiencies based on system’s results. Recently, a TDABC study compared the potential cost-savings in implementing what were judged to be cost-effective coronary artery bypass graft surgeries performed by top Indian hospitals in contrast to USA hospitals. The study concluded that the implementation of best practices observed in Indian hospitals would require modifications in regulations, family participation in the care process, and stronger support by hospital executives and clinicians [35].

At this time, the standardized framework for TDABC is supported by the literature and by the opinions of specialists with experience in applying TDABC in healthcare, although there is a lack of real-world full applications of the frameworks, and is therefore a research limitation. Our selection of experts was based on previously published TDABC studies, but we understand that with the application of the framework, methodological improvements from additional studies can be added to the framework. Also, the framework was designed in the context of healthcare business environment, even though the TDABC method was initially developed to increase the accuracy of unitary cost information in companies with high indirect costs in a variety of business sectors [37]. Once our standardized reporting framework establishes standardized reporting of healthcare studies, a similar process can be used to adapt it to any business sector.

Regarding the TDABC in Healthcare Consortium that has helped organize this work, this consensus statement helps introduce this innovative and global network. We believe that there are future opportunities for the development of international collaborative research and conferences for knowledge sharing to consolidate scientific advances for TDABC in the VBHC context.

## Conclusion

In conclusion, this Consensus Statement introduces a standardized framework, in a checklist format, for the application of TDABC to encourage researchers to perform higher quality TDABC costing studies that can be replicated, contributing to scaling of strategies that result in cost-savings in healthcare and other fields. Additionally, we believe that the standardized framework for healthcare and the creation of the TDABC Consortium can help connect and guide researchers, administrators, and other key decision-makers on how to quantitatively evaluate the impact of each strategy in projects designed to increase value in healthcare.

Future research should evaluate the benefits of the use of the TDABC standardized framework to report, in a scientific forum, TDABC projects developed in the context of healthcare business environment. This would help ensure the quality and interpretation of subsequent TDABC studies and help generate accurate cost information to support future decision-making processes.

### Supplementary Information

**Additional file 1.** Most cited TDABC research in healthcare articles.

## Data Availability

The tables presented along the entire article contains most data used to develop the article analysis. Additional datasets used during the current study are available from the corresponding author on reasonable request.

## Appendix

https://forms.gle/b38VGT8Frm4ED7yCA

## Abbreviations

TDABC: Time-driven Activity-based Costing
VBHC: Value-based Healthcare
CHEERS: Consolidated Health Economic Evaluation Reporting Standards
SPAQI: Society for Perioperative Assessment and Quality Improvement
ISPOR: International Society for Pharmacoeconomic and Outcomes Research
