# Supplementary material for: A standardized framework to evaluate the quality of studies using TDABC in healthcare: the TDABC in Healthcare Consortium Consensus Statement
Source: BMC Health Serv Res. 2020 Dec 1;20:1107. doi: 10.1186/s12913-020-05869-0 (PMC7706254; doi:10.1186/s12913-020-05869-0)
Supplement: Supplementary file 1 — Additional file 1. Most cited TDABC research in healthcare articles. [file 12913_2020_5869_MOESM1_ESM.docx]

Additional file 1 – Most cited TDABC research in healthcare articles

Database: Scopus

Search Strategy: [(Time-drive Activity-based Costing) or (TDABC)] and (Health*) in only Title, abstract or keywords.

| Articles Title | Number of citations on | Inclusion or not |
| --- | --- | --- |
| [Using time-driven activity-based costing to identify value improvement opportunities in healthcare](https://www.scopus.com/record/display.uri?eid=2-s2.0-84925021685&origin=resultslist&sort=cp-f&src=s&st1=TDABC+OR+%22time-driven+Activity-based+costing%22&st2=Health+OR+healthcare&nlo=&nlr=&nls=&sid=c2b5fc52b9688c1ea3761d65eb436a71&sot=b&sdt=cl&cluster=scopubstage%2c%22final%22%2ct%2bscosubtype%2c%22ar%22%2ct%2bscolang%2c%22English%22%2ct&sl=102&s=%28TITLE-ABS-KEY%28TDABC+OR+%22time-driven+Activity-based+costing%22%29+AND+TITLE-ABS-KEY%28Health+OR+healthcare%29%29&relpos=0&citeCnt=92&searchTerm=) (1) | 92 | Included in our analysis |
| [Time-driven activity-based costing in an outpatient clinic environment: Development, relevance and managerial impact](https://www.scopus.com/record/display.uri?eid=2-s2.0-69249214109&origin=resultslist&sort=cp-f&src=s&st1=TDABC+OR+%22time-driven+Activity-based+costing%22&st2=Health+OR+healthcare&nlo=&nlr=&nls=&sid=c2b5fc52b9688c1ea3761d65eb436a71&sot=b&sdt=cl&cluster=scopubstage%2c%22final%22%2ct%2bscosubtype%2c%22ar%22%2ct%2bscolang%2c%22English%22%2ct&sl=102&s=%28TITLE-ABS-KEY%28TDABC+OR+%22time-driven+Activity-based+costing%22%29+AND+TITLE-ABS-KEY%28Health+OR+healthcare%29%29&relpos=1&citeCnt=89&searchTerm=) (2) | 89 | Included in our analysis |
| Utilizing time-driven activity-based costing to understand the short- and long-term costs of treating localized, low-risk prostate cancer (3) | 75 | Included in our analysis |
| [Time-driven Activity-based Costing More Accurately Reflects Costs in Arthroplasty Surgery](https://www.scopus.com/record/display.uri?eid=2-s2.0-84952872200&origin=resultslist&sort=cp-f&src=s&st1=TDABC+OR+%22time-driven+Activity-based+costing%22&st2=Health+OR+healthcare&nlo=&nlr=&nls=&sid=c2b5fc52b9688c1ea3761d65eb436a71&sot=b&sdt=cl&cluster=scopubstage%2c%22final%22%2ct%2bscosubtype%2c%22ar%22%2ct%2bscolang%2c%22English%22%2ct&sl=102&s=%28TITLE-ABS-KEY%28TDABC+OR+%22time-driven+Activity-based+costing%22%29+AND+TITLE-ABS-KEY%28Health+OR+healthcare%29%29&relpos=3&citeCnt=67&searchTerm=) (4) | 67 | Included in our analysis |
| [Time-driven activity-based costing: A driver for provider engagement in costing activities and redesign initiatives](https://www.scopus.com/record/display.uri?eid=2-s2.0-84911471042&origin=resultslist&sort=cp-f&src=s&st1=TDABC+OR+%22time-driven+Activity-based+costing%22&st2=Health+OR+healthcare&nlo=&nlr=&nls=&sid=c2b5fc52b9688c1ea3761d65eb436a71&sot=b&sdt=cl&cluster=scopubstage%2c%22final%22%2ct%2bscosubtype%2c%22ar%22%2ct%2bscolang%2c%22English%22%2ct&sl=102&s=%28TITLE-ABS-KEY%28TDABC+OR+%22time-driven+Activity-based+costing%22%29+AND+TITLE-ABS-KEY%28Health+OR+healthcare%29%29&relpos=4&citeCnt=43&searchTerm=) (5) | 43 | Included in our analysis |
| [The Cost of Penicillin Allergy Evaluation](https://www.scopus.com/record/display.uri?eid=2-s2.0-85029705634&origin=resultslist&sort=cp-f&src=s&st1=TDABC+OR+%22time-driven+Activity-based+costing%22&st2=Health+OR+healthcare&nlo=&nlr=&nls=&sid=c2b5fc52b9688c1ea3761d65eb436a71&sot=b&sdt=cl&cluster=scopubstage%2c%22final%22%2ct%2bscosubtype%2c%22ar%22%2ct%2bscolang%2c%22English%22%2ct&sl=102&s=%28TITLE-ABS-KEY%28TDABC+OR+%22time-driven+Activity-based+costing%22%29+AND+TITLE-ABS-KEY%28Health+OR+healthcare%29%29&relpos=5&citeCnt=35&searchTerm=) (6) | 35 | Not-included: the article doesn’t detail the TDABC methodology |
| [Improving value with TDABC.](https://www.scopus.com/record/display.uri?eid=2-s2.0-84904561178&origin=resultslist&sort=cp-f&src=s&st1=TDABC+OR+%22time-driven+Activity-based+costing%22&st2=Health+OR+healthcare&nlo=&nlr=&nls=&sid=c2b5fc52b9688c1ea3761d65eb436a71&sot=b&sdt=cl&cluster=scopubstage%2c%22final%22%2ct%2bscosubtype%2c%22ar%22%2ct%2bscolang%2c%22English%22%2ct&sl=102&s=%28TITLE-ABS-KEY%28TDABC+OR+%22time-driven+Activity-based+costing%22%29+AND+TITLE-ABS-KEY%28Health+OR+healthcare%29%29&relpos=6&citeCnt=35&searchTerm=) (7) | 34 | Included in our analysis |
| [Measuring the cost of care in benign prostatic hyperplasia using time-driven activity-based costing (TDABC)](https://www.scopus.com/record/display.uri?eid=2-s2.0-84941572753&origin=resultslist&sort=cp-f&src=s&st1=TDABC+OR+%22time-driven+Activity-based+costing%22&st2=Health+OR+healthcare&nlo=&nlr=&nls=&sid=c2b5fc52b9688c1ea3761d65eb436a71&sot=b&sdt=cl&cluster=scopubstage%2c%22final%22%2ct%2bscosubtype%2c%22ar%22%2ct%2bscolang%2c%22English%22%2ct&sl=102&s=%28TITLE-ABS-KEY%28TDABC+OR+%22time-driven+Activity-based+costing%22%29+AND+TITLE-ABS-KEY%28Health+OR+healthcare%29%29&relpos=7&citeCnt=34&searchTerm=) (8) | 33 | Included in our analysis |
| [Costs of complications after colorectal cancer surgery in the Netherlands: Building the business case for hospitals](https://www.scopus.com/record/display.uri?eid=2-s2.0-84943390605&origin=resultslist&sort=cp-f&src=s&st1=TDABC+OR+%22time-driven+Activity-based+costing%22&st2=Health+OR+healthcare&nlo=&nlr=&nls=&sid=c2b5fc52b9688c1ea3761d65eb436a71&sot=b&sdt=cl&cluster=scopubstage%2c%22final%22%2ct%2bscosubtype%2c%22ar%22%2ct%2bscolang%2c%22English%22%2ct&sl=102&s=%28TITLE-ABS-KEY%28TDABC+OR+%22time-driven+Activity-based+costing%22%29+AND+TITLE-ABS-KEY%28Health+OR+healthcare%29%29&relpos=8&citeCnt=33&searchTerm=) (9) | 32 | Not-included: the article doesn’t detail the TDABC methodology |
| [Elimination of Routine Contact Precautions for Endemic Methicillin-Resistant Staphylococcus aureus and Vancomycin-Resistant Enterococcus: A Retrospective Quasi-Experimental Study](https://www.scopus.com/record/display.uri?eid=2-s2.0-84992437425&origin=resultslist&sort=cp-f&src=s&st1=TDABC+OR+%22time-driven+Activity-based+costing%22&st2=Health+OR+healthcare&nlo=&nlr=&nls=&sid=c2b5fc52b9688c1ea3761d65eb436a71&sot=b&sdt=cl&cluster=scopubstage%2c%22final%22%2ct%2bscosubtype%2c%22ar%22%2ct%2bscolang%2c%22English%22%2ct&sl=102&s=%28TITLE-ABS-KEY%28TDABC+OR+%22time-driven+Activity-based+costing%22%29+AND+TITLE-ABS-KEY%28Health+OR+healthcare%29%29&relpos=9&citeCnt=32&searchTerm=) (10) | 32 | Not-included: the article doesn’t detail the TDABC methodology |
| [How Cleveland Clinic used TDABC to improve value.](https://www.scopus.com/record/display.uri?eid=2-s2.0-84904560771&origin=resultslist&sort=cp-f&src=s&st1=TDABC+OR+%22time-driven+Activity-based+costing%22&st2=Health+OR+healthcare&nlo=&nlr=&nls=&sid=c2b5fc52b9688c1ea3761d65eb436a71&sot=b&sdt=cl&cluster=scopubstage%2c%22final%22%2ct%2bscosubtype%2c%22ar%22%2ct%2bscolang%2c%22English%22%2ct&sl=102&s=%28TITLE-ABS-KEY%28TDABC+OR+%22time-driven+Activity-based+costing%22%29+AND+TITLE-ABS-KEY%28Health+OR+healthcare%29%29&relpos=10&citeCnt=32&searchTerm=) (11) | 32 | Included in our analysis |
| [Measuring the value of process improvement initiatives in a preoperative assessment center using time-driven activity-based costing](https://www.scopus.com/record/display.uri?eid=2-s2.0-84890143210&origin=resultslist&sort=cp-f&src=s&st1=TDABC+OR+%22time-driven+Activity-based+costing%22&st2=Health+OR+healthcare&nlo=&nlr=&nls=&sid=c2b5fc52b9688c1ea3761d65eb436a71&sot=b&sdt=cl&cluster=scopubstage%2c%22final%22%2ct%2bscosubtype%2c%22ar%22%2ct%2bscolang%2c%22English%22%2ct&sl=102&s=%28TITLE-ABS-KEY%28TDABC+OR+%22time-driven+Activity-based+costing%22%29+AND+TITLE-ABS-KEY%28Health+OR+healthcare%29%29&relpos=11&citeCnt=32&searchTerm=) (12) | 32 | Included in our analysis |
| [Hospital costs of complications after esophagectomy for cancer](https://www.scopus.com/record/display.uri?eid=2-s2.0-85010790000&origin=resultslist&sort=cp-f&src=s&st1=TDABC+OR+%22time-driven+Activity-based+costing%22&st2=Health+OR+healthcare&nlo=&nlr=&nls=&sid=c2b5fc52b9688c1ea3761d65eb436a71&sot=b&sdt=cl&cluster=scopubstage%2c%22final%22%2ct%2bscosubtype%2c%22ar%22%2ct%2bscolang%2c%22English%22%2ct&sl=102&s=%28TITLE-ABS-KEY%28TDABC+OR+%22time-driven+Activity-based+costing%22%29+AND+TITLE-ABS-KEY%28Health+OR+healthcare%29%29&relpos=12&citeCnt=30&searchTerm=) (13) | 30 | Not-included: the article doesn’t detail the TDABC methodology |
| [Stereotactic body radiotherapy for lung cancer: How much does it really cost?](https://www.scopus.com/record/display.uri?eid=2-s2.0-84938294097&origin=resultslist&sort=cp-f&src=s&st1=TDABC+OR+%22time-driven+Activity-based+costing%22&st2=Health+OR+healthcare&nlo=&nlr=&nls=&sid=c2b5fc52b9688c1ea3761d65eb436a71&sot=b&sdt=cl&cluster=scopubstage%2c%22final%22%2ct%2bscosubtype%2c%22ar%22%2ct%2bscolang%2c%22English%22%2ct&sl=102&s=%28TITLE-ABS-KEY%28TDABC+OR+%22time-driven+Activity-based+costing%22%29+AND+TITLE-ABS-KEY%28Health+OR+healthcare%29%29&relpos=13&citeCnt=28&searchTerm=) (14) | 28 | Not-included: the article doesn’t detail the TDABC methodology |
| [Improving patient-level costing in the English and the German 'DRG' system](https://www.scopus.com/record/display.uri?eid=2-s2.0-84874314168&origin=resultslist&sort=cp-f&src=s&st1=TDABC+OR+%22time-driven+Activity-based+costing%22&st2=Health+OR+healthcare&nlo=&nlr=&nls=&sid=c2b5fc52b9688c1ea3761d65eb436a71&sot=b&sdt=cl&cluster=scopubstage%2c%22final%22%2ct%2bscosubtype%2c%22ar%22%2ct%2bscolang%2c%22English%22%2ct&sl=102&s=%28TITLE-ABS-KEY%28TDABC+OR+%22time-driven+Activity-based+costing%22%29+AND+TITLE-ABS-KEY%28Health+OR+healthcare%29%29&relpos=14&citeCnt=28&searchTerm=) (15) | 28 | Not-included: the article doesn’t detail the TDABC methodology |
| [Administrative costs associated with physician billing and insurance-related activities at an academic health care system](https://www.scopus.com/record/display.uri?eid=2-s2.0-85042255566&origin=resultslist&sort=cp-f&src=s&st1=TDABC+OR+%22time-driven+Activity-based+costing%22&st2=Health+OR+healthcare&nlo=&nlr=&nls=&sid=c2b5fc52b9688c1ea3761d65eb436a71&sot=b&sdt=cl&cluster=scopubstage%2c%22final%22%2ct%2bscosubtype%2c%22ar%22%2ct%2bscolang%2c%22English%22%2ct&sl=102&s=%28TITLE-ABS-KEY%28TDABC+OR+%22time-driven+Activity-based+costing%22%29+AND+TITLE-ABS-KEY%28Health+OR+healthcare%29%29&relpos=15&citeCnt=27&searchTerm=) (16) | 27 | Included in our analysis |
| [Measuring radiology's value in time saved](https://www.scopus.com/record/display.uri?eid=2-s2.0-84926231947&origin=resultslist&sort=cp-f&src=s&st1=TDABC+OR+%22time-driven+Activity-based+costing%22&st2=Health+OR+healthcare&nlo=&nlr=&nls=&sid=c2b5fc52b9688c1ea3761d65eb436a71&sot=b&sdt=cl&cluster=scopubstage%2c%22final%22%2ct%2bscosubtype%2c%22ar%22%2ct%2bscolang%2c%22English%22%2ct&sl=102&s=%28TITLE-ABS-KEY%28TDABC+OR+%22time-driven+Activity-based+costing%22%29+AND+TITLE-ABS-KEY%28Health+OR+healthcare%29%29&relpos=16&citeCnt=27&searchTerm=) (17) | 27 | Not-included: the article doesn’t detail the TDABC methodology |
| [Evaluation of Delivery Costs for External Beam Radiation Therapy and Brachytherapy for Locally Advanced Cervical Cancer Using Time-Driven Activity-Based Costing](https://www.scopus.com/record/display.uri?eid=2-s2.0-85032206928&origin=resultslist&sort=cp-f&src=s&st1=TDABC+OR+%22time-driven+Activity-based+costing%22&st2=Health+OR+healthcare&nlo=&nlr=&nls=&sid=c2b5fc52b9688c1ea3761d65eb436a71&sot=b&sdt=cl&cluster=scopubstage%2c%22final%22%2ct%2bscosubtype%2c%22ar%22%2ct%2bscolang%2c%22English%22%2ct&sl=102&s=%28TITLE-ABS-KEY%28TDABC+OR+%22time-driven+Activity-based+costing%22%29+AND+TITLE-ABS-KEY%28Health+OR+healthcare%29%29&relpos=17&citeCnt=25&searchTerm=) (18) | 25 | Included in our analysis |
| [Time-driven activity based costing of total knee replacement surgery at a London teaching hospital](https://www.scopus.com/record/display.uri?eid=2-s2.0-84973408138&origin=resultslist&sort=cp-f&src=s&st1=TDABC+OR+%22time-driven+Activity-based+costing%22&st2=Health+OR+healthcare&nlo=&nlr=&nls=&sid=c2b5fc52b9688c1ea3761d65eb436a71&sot=b&sdt=cl&cluster=scopubstage%2c%22final%22%2ct%2bscosubtype%2c%22ar%22%2ct%2bscolang%2c%22English%22%2ct&sl=102&s=%28TITLE-ABS-KEY%28TDABC+OR+%22time-driven+Activity-based+costing%22%29+AND+TITLE-ABS-KEY%28Health+OR+healthcare%29%29&relpos=22&citeCnt=24&searchTerm=) (19) | 24 | Included in our analysis |
| Time-driven activity-based costing of multivessel coronary artery bypass grafting across national boundaries to identify improvement opportunities: study protocol. (20) | 24 | Included in our analysis |
| [Nationwide Outcomes Measurement in Colorectal Cancer Surgery: Improving Quality and Reducing Costs Presented at the European Society of Surgical Oncology 34th Congress, Liverpool, United Kingdom, October 2014.](https://www.scopus.com/record/display.uri?eid=2-s2.0-84951755000&origin=resultslist&sort=cp-f&src=s&st1=TDABC+OR+%22time-driven+Activity-based+costing%22&st2=Health+OR+healthcare&nlo=&nlr=&nls=&sid=c2b5fc52b9688c1ea3761d65eb436a71&sot=b&sdt=cl&cluster=scopubstage%2c%22final%22%2ct%2bscosubtype%2c%22ar%22%2ct%2bscolang%2c%22English%22%2ct&sl=102&s=%28TITLE-ABS-KEY%28TDABC+OR+%22time-driven+Activity-based+costing%22%29+AND+TITLE-ABS-KEY%28Health+OR+healthcare%29%29&relpos=21&citeCnt=24&searchTerm=) (21) | 24 | Not-included: the article doesn’t detail the TDABC methodology |
| [Determining the True Cost to Deliver Total Hip and Knee Arthroplasty Over the Full Cycle of Care: Preparing for Bundling and Reference-Based Pricing](https://www.scopus.com/record/display.uri?eid=2-s2.0-84953839620&origin=resultslist&sort=cp-f&src=s&st1=TDABC+OR+%22time-driven+Activity-based+costing%22&st2=Health+OR+healthcare&nlo=&nlr=&nls=&sid=c2b5fc52b9688c1ea3761d65eb436a71&sot=b&sdt=cl&cluster=scopubstage%2c%22final%22%2ct%2bscosubtype%2c%22ar%22%2ct%2bscolang%2c%22English%22%2ct&sl=102&s=%28TITLE-ABS-KEY%28TDABC+OR+%22time-driven+Activity-based+costing%22%29+AND+TITLE-ABS-KEY%28Health+OR+healthcare%29%29&relpos=20&citeCnt=24&searchTerm=) (22) | 24 | Not-included: the article doesn’t detail the TDABC methodology |
| [Analysis of Direct Costs of Outpatient Arthroscopic Rotator Cuff Repair](https://www.scopus.com/record/display.uri?eid=2-s2.0-84988499971&origin=resultslist&sort=cp-f&src=s&st1=TDABC+OR+%22time-driven+Activity-based+costing%22&st2=Health+OR+healthcare&nlo=&nlr=&nls=&sid=c2b5fc52b9688c1ea3761d65eb436a71&sot=b&sdt=cl&cluster=scopubstage%2c%22final%22%2ct%2bscosubtype%2c%22ar%22%2ct%2bscolang%2c%22English%22%2ct&sl=102&s=%28TITLE-ABS-KEY%28TDABC+OR+%22time-driven+Activity-based+costing%22%29+AND+TITLE-ABS-KEY%28Health+OR+healthcare%29%29&relpos=24&citeCnt=22&searchTerm=) (23) | 22 | Not-included: the article doesn’t detail the TDABC methodology |
| [Defining the value framework for prostate brachytherapy using patient-centered outcome metrics and time-driven activity-based costing](https://www.scopus.com/record/display.uri?eid=2-s2.0-84964550134&origin=resultslist&sort=cp-f&src=s&st1=TDABC+OR+%22time-driven+Activity-based+costing%22&st2=Health+OR+healthcare&nlo=&nlr=&nls=&sid=c2b5fc52b9688c1ea3761d65eb436a71&sot=b&sdt=cl&cluster=scopubstage%2c%22final%22%2ct%2bscosubtype%2c%22ar%22%2ct%2bscolang%2c%22English%22%2ct&sl=102&s=%28TITLE-ABS-KEY%28TDABC+OR+%22time-driven+Activity-based+costing%22%29+AND+TITLE-ABS-KEY%28Health+OR+healthcare%29%29&relpos=27&citeCnt=21&searchTerm=) (24) | 21 | Included in our analysis |
| [Time-driven activity-based costing: A dynamic value assessment model in pediatric appendicitis](https://www.sciencedirect.com/science/article/pii/S0022346817301811?casa_token=S7nkkigmMAEAAAAA:E_iuKyK74aCGA7cb8JvZSvyShRESPou9nrgw739y0p0KM4E4cP7SghdqYb8GWWOqz6BBmbJtyw) (25) | 21 | Included in our analysis |

TDABC: time-driven, activity-based costing

1. Kaplan RS, Witkowski M, Abbott M, Barboza Guzman A, Higgins LD, Meara JG, et al. Using Time-Driven Activity-Based Costing to Identify Value Improvement Opportunities in Healthcare. Journal of Healthcare Management. 2014;59(6).

2. Demeere N, Stouthuysen K, Roodhooft F. Time-driven activity-based costing in an outpatient clinic environment: Development, relevance and managerial impact. Health Policy. 2009;92:296–304.

3. Laviana AA, Ilg AM, Veruttipong D, Tan HJ, Burke MA, Niedzwiecki DR, et al. Utilizing time-driven activity-based costing to understand the short- and long-term costs of treating localized, low-risk prostate cancer. Cancer. 2016;122(3):447–55.

4. Akhavan S, Ward L, Bozic KJ. Time-driven Activity-based Costing More Accurately Reflects Costs in Arthroplasty Surgery. Clinical Orthopaedics and Related Research. 2016;474(1).

5. McLaughlin N, Burke MA, Setlur NP, Niedzwiecki DR, Kaplan AL, Saigal C, et al. Time-driven activity-based costing: a driver for provider engagement in costing activities and redesign initiatives. Neurosurgical focus. 2014;

6. Blumenthal KG, Li Y, Banerji A, Yun BJ, Long AA, Walensky RP. The cost of penicillin allergy evaluation. The Journal of Allergy and Clinical Immunology: In Practice. 2018;6(3):1019–27.

7. Kaplan RS. Improving value with TDABC. 2014.

8. Kaplan AL, Agarwal N, Setlur NP, Tan HJ, Niedzwiecki D, McLaughlin N, et al. Measuring the cost of care in benign prostatic hyperplasia using time-driven activity-based costing (TDABC). Healthcare. 2015 Mar;3(1):43–8.

9. Govaert J, Fiocco M, van Dijk W, Scheffer A, de Graaf E, Tollenaar R, et al. Costs of complications after colorectal cancer surgery in the Netherlands: building the business case for hospitals. European Journal of Surgical Oncology (EJSO). 2015;41(8):1059–67.

10. Martin EM, Russell D, Rubin Z, Humphries R, Grogan TR, Elashoff D, et al. Elimination of routine contact precautions for endemic methicillin-resistant Staphylococcus aureus and vancomycin-resistant Enterococcus: a retrospective quasi-experimental study. infection control & hospital epidemiology. 2016;37(11):1323–30.

11. Donovan CJ, Hopkins M, Kimmel BM, Koberna S, Montie CA. How Cleveland Clinic used TDABC to improve value. Healthcare Financial Management. 2014;68(6):84–9.

12. French KE, Albright HW, Frenzel JC, Incalcaterra JR, Rubio AC, Jones JF, et al. Measuring the value of process improvement initiatives in a preoperative assessment center using time-driven activity-based costing. Healthcare. 2013 Dec;1(3–4):136–42.

13. Goense L, Van Dijk W, Govaert JA, van Rossum PS, Ruurda JP, van Hillegersberg R. Hospital costs of complications after esophagectomy for cancer. European Journal of Surgical Oncology (EJSO). 2017;43(4):696–702.

14. Lievens Y, Obyn C, Mertens A-S, Van Halewyck D, Hulstaert F. Stereotactic body radiotherapy for lung cancer: how much does it really cost? Journal of Thoracic Oncology. 2015;10(3):454–61.

15. Vogl M. Improving patient-level costing in the English and the German ‘DRG’system. Health Policy. 2013;109(3):290–300.

16. Tseng P, Kaplan RS, Richman BD, Shah MA, Schulman KA. Administrative costs associated with physician billing and insurance-related activities at an academic health care system. Jama. 2018;319(7):691–7.

17. Lee CI, Enzmann DR. Measuring radiology’s value in time saved. Journal of the American College of Radiology. 2012;9(10):713–7.

18. Bauer-Nilsen K, Hill C, Trifiletti DM, Libby B, Lash DH, Lain M, et al. Evaluation of delivery costs for external beam radiation therapy and brachytherapy for locally advanced cervical cancer using time-driven activity-based costing. International Journal of Radiation Oncology* Biology* Physics. 2018;100(1):88–94.

19. Chen A, Sabharwal S, Akhtar K, Makaram N, Gupte CM. Time-driven activity based costing of total knee replacement surgery at a London teaching hospital. The Knee. 2015 Dec;22(6):640–5.

20. Erhun F, Mistry B, Platchek T, Milstein A, Narayanan VG, Kaplan RS. Time-driven activity-based costing of multivessel coronary artery bypass grafting across national boundaries to identify improvement opportunities: study protocol. BMJ Open. 2015 Aug;5(8):e008765.

21. Govaert JA, van Dijk WA, Fiocco M, Scheffer AC, Gietelink L, Wouters MW, et al. Nationwide outcomes measurement in colorectal cancer surgery: improving quality and reducing costs. Journal of the American College of Surgeons. 2016;222(1):19–29.

22. DiGioia III AM, Greenhouse PK, Giarrusso ML, Kress JM. Determining the true cost to deliver total hip and knee arthroplasty over the full cycle of care: preparing for bundling and reference-based pricing. The Journal of Arthroplasty. 2016;31(1):1–6.

23. Narvy SJ, Ahluwalia A, Vangsness C. Analysis of direct costs of outpatient arthroscopic rotator cuff repair. Am J Orthop (Belle Mead NJ). 2016;45(1):E7–11.

24. Thaker NG, Pugh TJ, Mahmood U, Choi S, Spinks TE, Martin NE, et al. Defining the value framework for prostate brachytherapy using patient-centered outcome metrics and time-driven activity-based costing. Brachytherapy. 2016 May;15(3):274–82.

25. Yangyang RY, Abbas PI, Smith CM, Carberry KE, Ren H, Patel B, et al. Time-driven activity-based costing: A dynamic value assessment model in pediatric appendicitis. Journal of pediatric surgery. 2017;52(6):1045–9.
